# Distributed lag non-linear models

**DOI:** 10.1002/sim.3940

**Published:** 2010-05-07

**Authors:** A Gasparrini, B Armstrong, M G Kenward

**Affiliations:** a Public Health and Policy Department, London School of Hygiene and Tropical MedicineKeppel Street, London W1C 7HT, U.K.; b Epidemiology and Population Health Department, London School of Hygiene and Tropical MedicineLondon, U.K.

**Keywords:** distributed lag, time series, smoothing, delayed effects

## Abstract

Environmental stressors often show effects that are delayed in time, requiring the use of statistical models that are flexible enough to describe the additional time dimension of the exposure–response relationship. Here we develop the family of distributed lag non-linear models (DLNM), a modelling framework that can simultaneously represent non-linear exposure–response dependencies and delayed effects. This methodology is based on the definition of a ‘cross-basis’, a bi-dimensional space of functions that describes simultaneously the shape of the relationship along both the space of the predictor and the lag dimension of its occurrence. In this way the approach provides a unified framework for a range of models that have previously been used in this setting, and new more flexible variants. This family of models is implemented in the package dlnm within the statistical environment R. To illustrate the methodology we use examples of DLNMs to represent the relationship between temperature and mortality, using data from the National Morbidity, Mortality, and Air Pollution Study (NMMAPS) for New York during the period 1987–2000. Copyright © 2010 John Wiley & Sons, Ltd.

## 1. Introduction

Sometimes the effect of a specific exposure event is not limited to the period when it is observed, but it is *delayed* in time. This introduces the problem of modelling the relationship between an exposure occurrence and a sequence of future outcomes, specifying the distribution of the effects at different times after the event (defined *lags*). Ultimately, this step requires the definition of the additional lag dimension of an exposure–response relationship, describing the *time structure* of the effect.

This situation occurs frequently when assessing the short-term effects of environmental stressors: several time-series studies have reported that the exposure to high levels of air pollution or extreme temperatures affect health for a period lasting some days after its occurrence [1, 2]. Furthermore, the complexity increases in the presence of so-called ‘harvesting': the phenomenon that arises when a stressor affects mainly a pool of frail individuals, whose events are only brought forward by a brief period of time by the effect of exposure [3, 4]. For non-recurrent outcomes, the depletion of the pool following a stress results in some reduction of cases few days later, thereby reducing the overall long-term impact. For both these reasons, the estimate of the effect depends on the appropriate specification of the lag dimension of the dependency, defining models flexible enough to represent simultaneously the exposure-response relationship and its temporal structure.

Among the various methods that have been proposed to deal with delayed effects, a major role is played by *distributed lag models* (DLM), recently used to quantify the health effect and assess the presence of harvesting in air pollution and temperature studies [2, 5, 6]. The main advantage of this method is that it allows the model to contain a detailed representation of the time-course of the exposure–response relationship, which in turn provides an estimate of the overall effect in the presence of delayed contributions or harvesting.

While conventional DLMs are suitable for describing the lag structure of linear effects, they show some limitations when used to represent non-linear relationships. We propose a solution, to relax further the assumptions on the shape of the relationship and extend this methodology to *distributed lag non-linear models* (DLNM), a family of models which can describe, in a flexible way, effects that vary simultaneously both along the space of the predictor and in the lag dimension of its occurrence. In this way the class of DLNMs also provides a unifying framework for existing simpler methods.

DLNMs have been previously described only briefly in epidemiological terms [7]: the aim of this paper is to develop this method rigorously, and to describe implementation in the specifically written package dlnm included in the statistical software R [8], providing an illustrative example of its application using a real data set. In Section 2 we briefly describe the basic model used in time series analysis and introduce the idea of basis as a general way to describe a non-linear relationship between a predictor and a response. In Section 3 we outline the additional complexity of effects delayed in time and provide a general representation of simple DLMs. In Section 4 we use the results obtained in the previous sections to define the general framework of DLNMs which includes all the models previously described as special cases. An application of this methodology to modelling the effect of temperature on mortality for New York is illustrated in Section 5. In Section 6 we provide some discussion and propose possible further developments.

## 2. The basic model

### 2.1. A general representation

A general model representation to describe the time series of outcomes *Y_t_* with *t* = 1,…, *n* is given by


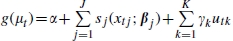
(1)

where μ ≡ *E(Y), g* is a monotonic link function and *Y* is assumed to arise from a distribution belonging to the exponential family [9, 10]. The functions *s_j_* denote smoothed relationships between the variables *x_j_* and the linear predictor, defined by the parameter vectors β_*j*_. The variables *u_k_* include other predictors with linear effects specified by the related coefficients γ_*k*_. The functions *s_j_* might be also specified through non-parametric methods based on generalized additive models [11, 12]. However, in the present development we rely on a completely parametric approach.

In time series analyses of environmental factors the outcomes *Y_t_* are commonly daily counts, assumed to originate from a so-called overdispersed Poisson distribution with *E(Y)* = μ, V(*Y*) = φμ, and a canonical log-link in (1). These studies have taken advantage of the substantial improvements, during the last years, of statistical methods to quantify the short-term effects of air pollution [13, 14]. Usually these include a smooth function of time to capture the effect of confounders changing slowly over time, expressed as seasonality or long-time trends. Non-linear effects of metereological factors such as temperature and humidity are included as well. Categorical variables such as days of the week or age groups are modelled as factors. Although air pollution is commonly described by a linear relationship, this assumption may be relaxed in order to assess non-linear effects.

Here we focus on a general function *s* specifying the potentially non-linear and delayed effect of the predictor *x*, often referring, without loss of generality, to air pollution or temperature.

### 2.2. Basis functions

The relationship between *x* and *g*(μ) is represented by *s(x)*, which is included in the linear predictor of a generalized linear model as a sum of linear terms. This can be done through the choice of a *basis*, a space of functions of which we believe *s* to be an element [12]. The related *basis functions* comprise a set of completely known transformations of the original variable *x* that generate a new set of variables, termed *basis variables*. The complexity of the estimated relationship depends on the type of basis and its dimension.

Several different basis functions have been used to describe the potentially non-linear health effects of environmental factors, the choice depending on the assumptions about the shape of the relationship, the degree of approximation required by the specific purposes of the investigation, and interpretational issues. Among completely parametric methods, the main choices typically rely on functions describing smooth curves, such as polynomials or spline functions [15], or on the use of a linear threshold parameterization, represented by a truncated linear function (*x* − *k*)_+_ which equals (*x* − *k*) when *x* > *k* and 0 otherwise [16].

A general representation of the simple models described above is given by


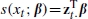
(2)

with **z**_*t*_. as the *t*th row of the *n* × *v_x_*, basis matrix **Z**, obtained by the application of the basis functions to the original vector of exposures **x. Z** can be then included in the design matrix of the model in (1) in order to estimate the related unknown parameters β defining the shape of the relationship.

## 3. Delayed effects

### 3.1. An additional dimension

In the presence of delayed effects, the outcome at a given time *t* may be explained in terms of past exposures *x*_*t*−ℓ_, with ℓ as the *lag*, representing the period elapsed between the exposure and the response. A comparatively simple approach is to apply a transformation to the original vector of ordered exposures **x**, deriving the *n* × (*L* +1) matrix **Q**, such as


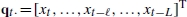
(3)

with *L* defining the *maximum lag* and **q**.i ≡ **x** (the first column of **Q**). We can also define ℓ = [0, …, ℓ, …, *L*]^T^ as vector of lags corresponding to the *L* + 1 columns of **Q**.

This step specifies the additional lag dimension of the exposure-response relationship. Ultimately, the aim of the modelling framework proposed here is to simultaneously describe the dependency along two dimensions: the usual predictor space and in the new lag dimension.

### 3.2. Distributed lag models

When a linear relationship is assumed, the delayed effects can be naturally described by distributed lag models (DLM). This methodology allows the effect of a single exposure event to be distributed over a specific period of time, using several parameters to explain the contributions at different lags. These models have been extensively used to assess the lagged effects of environmental factors. The simplest formulation is an *unconstrained* DLM, specified by the inclusion of a parameter for each lag [5, 17]. Unfortunately, the precision of the estimates for the effects at specific lags is often very poor, due to the high correlation between exposures in adjacent days and the resulting collinearity in the model [1]. To gain more precision in the estimate of the distributed lag curve, it is possible to impose some constraints, for example assuming a constant effect within lag intervals [18], or describing a smooth curve using continuous functions such as polynomials [5, 19] or splines [6]. A simple model with the moving average of the exposures in the previous *L* days as a predictor can be considered as a special case of a DLM: such a model has been extensively used in the field of air pollution epidemiology [20] and sometimes used as well to quantify the effects of temperatures [21].

The algebraic notation for this class of models has only been given previously for polynomial DLMs [5]. Using the development provided in Sections 2.2 and 3.1, it is possible to formulate a simpler and general definition of DLM, in which the shape of the distributed effects along lags is specified by a proper basis. In matrix notation


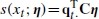
(4)

where **C** is an (*L* +1) × *v*_ℓ_ matrix of basis variables derived from the application of the specific basis functions to the lag vector ℓ, and η a vector of unknown parameters. The addition of the supplementary dimension in (3) provides a structure for the application of the basis matrix **C**, in order to describe the effects of lagged exposures. All the different DLMs described above can be derived from (4), by specifying the correspondent basis matrix: **C** ≡ **1** (a vector of ones) for the moving average model, **C** ≡ **I** (an identity matrix) for the unconstrained DLM, or **C** defined as a series of polynomial or splines functions of ℓ for DLMs describing the effect as a smoothed curve along lags. From (4) we can define


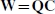
(5)

with **W** the matrix of the *v*_ℓ_ transformed variables that are included in the design matrix to allow estimation of the parameters η. The interpretation of the estimated parameters 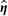 is aided by construction from them of the implied linear effects β at each lag, following:


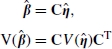
(6)

Here the choice of the basis to derive C can be considered as the application of a constraint to the shape of the distributed lag curve described by 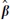.

Despite the specification of the basis functions in (4) being slightly different to that in (2), i.e. being applied to the vector ℓ instead of the exposure series **x** itself, their goal is conceptually similar to describe the shape of the relationship, the former along distributed lags and the latter in the space of *x*.

## 4. Distributed lag non-linear models

As described in Sections 2 and 3, there are well-developed methods to describe flexible exposure–response relationships for simple lag models, or alternatively flexible DLMs for simple linear effects, but rarely are these two components modelled simultaneously. Extensions to describe non-linear effects have been proposed, using a piecewise parameterization or polynomials, for which a DLM can be constructed by applying the constraint matrix C to each term of a threshold [22] or piecewise function [23] or to the linear and quadratic terms [2], respectively. Nonetheless, these methods remain somewhat limited in their ability to describe this complex dependency.

A useful generalization is achieved through the generation of a new model framework which can describe non-linear relationships both in the space of the predictor and along lags, leading to the family of DLNM.

### 4.1. The concept of cross-basis

While the algebraic notation of DLNMs can be quite complex, involving three-dimensional arrays, the basic concept, which rests on the definition of a *cross-basis*, is straightforward. Extending the idea of basis described in Section 2, a cross-basis can be pictured as a bi-dimensional space of functions describing simultaneously the shape of the relationship along *x* and its distributed lag effects. Choosing a cross-basis amounts to choosing two sets of basis functions, which will be combined to generate the *cross-basis functions*.

### 4.2. The algebra of DLNM

To model the shape of the relationship in each of the two spaces we are considering, we need to apply simultaneously the two transformations described in Sections 2 and 3. First, as in (2), we choose a basis for **x** to define the dependency in the space of the predictor, specifying **Z**. Then we create the additional lag dimension, as in (3), for each one of the derived basis variables of **x** stored in **Z**. This produces a *n* × *v_x_* × (*L* + 1) array **Ṙ**, which represents the lagged occurrences of each of the basis variables of **x**. The construction is symmetric, in the sense that the order of the two transformations can be reversed, applying the basis functions directly to each column of the matrix **Q**.

Defining **C**, the matrix of basis variables for ℓ seen in (4), a DLNM can then be specified by


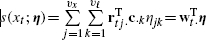
(7)

where **r**_*tj.*_ is the vector of lagged exposures for the time *t* transformed through the basis function *j*. The vector **w**_*t*_. is obtained by applying the *v_x_* · *v*_ℓ_ cross-basis functions to *x_t_*, similarly to (5). We keep the same notation to emphasize the fact that the DLM specified in (4) is a special case of the more general DLNM in (7). To reach a compact formula for **W** of a similar form to (5), we need to present it as a tensor product. Defining *P_i, j_* as the operator permuting the indexes *i* and *j* of an array and assuming a generic *i* × *j* matrix as a *i* × *j* × 1 array, it follows that


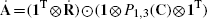
(8)

with **1** indicating vectors of ones with appropriate dimensions. The symbols ⊗ and ⊙ represent the Kronecker and Hadamard products, respectively. The *n* × (*v_x_* · *v_l_*) × (*L* + 1) array **Ȧ** is then re-arranged, summing along the third dimension of lags to obtain the final matrix of cross-basis functions **W**. The equation in (8) is a modified version of the formula used to implement smoothing on a multidimensional grid through tensor product bases [24, 25]. The main difference in the cross-basis approach lies in the dimensions considered in the model. While the original method provides a framework to describe a smooth surface in the space of two distinct variables, the DLNM expresses simultaneously the effects in the space of a variable and in its lag dimension.

### 4.3. Interpreting a DLNM

Despite its complex parameterization, estimation of and inference about the parameters of a DLNM raise no more problems than any other generalized linear model, and can be carried out with common statistical softwares after the cross-basis variables have been specified. Nonetheless, while the interpretation of the simpler DLM in (4) is straightforward, consisting in reporting the estimated linear effects 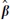 in (6) for each lag, the results of a more complex DLNM with smoothed non-linear dependencies are harder to summarize. One solution is to build a grid of predictions for each lag and for suitable values of exposure, using three-dimensional plots to provide an overall picture of the effects varying along the two dimensions.

Given a vector **x**^*p*^ of the *m* exposure values used for prediction and the resultant *m* × *v_x_* matrix **Z**^*p*^, the corresponding *m* × *v_x_* × (*L* + 1) array **Ṙ**^*p*^ can be derived by repeating the matrix **Z**^*p*^ *L* + 1 times along the dimension of the lags. The computation of **Ṙ**^*p*^ is slightly different than for the array **Ṙ** used in the estimation process in (7). In this case the interest lies in the prediction of the effects at each lag given an exposure, not in the temporal sequence of the exposures themselves. The final array **Ȧ**^*p*^ follows simply substituting **r**_*tj*._. with 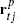 in (7) or **Ṙ** with **Ṙ**^*p*^ in (8).

The prediction grid, expressed with the *m* × (*L* + 1) matrix of predicted effects **E** and related matrix of associated standard errors **E**^*sd*^, can be derived using the vector of estimated coefficients 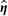, computed from the model fitted including the matrix of cross-basis functions **W**. For each lag ℓ


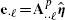
(9)

and, given 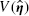 the variance-covariance matrix of the estimated coefficients


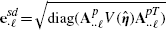
(10)

This grid is useful to compute the estimates of the effects by exposure at lag ℓ_*p*_ or by lag at exposure *x_p_*, simply taking **e**_.ℓ_*p*__ and **e***_x_x__*, respectively.

Finally, an estimate of the overall effect can be computed by summing all the contributions at different lags. The vector e_tot_ and associated standard errors 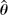, obtained summing the contributions at each lag, specify the effects by exposure over the whole lag period. They are obtained from


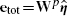
(11)

and


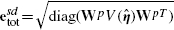
(12)

## 5. An application

### 5.1. Data and model choices

We apply DLNMs to investigate the effect of temperature on overall mortality for the city of New York, during the period 1987–2000. The data set is taken from the National Morbidity, Mortality, and Air Pollution Study (NMMAPS) [26], available publicly through the Internet-based Health and Air Pollution Surveillance System website (http://www.ihapss.jhsph.edu). It includes 5114 daily observations of overall and cause-specific mortality, weather and pollution data.

The analysis is based on the model in (1), fitted through a generalized linear model with quasi-Poisson family, with the following choices regarding the control of confounders: natural cubic splines of time with 7 degrees of freedom (df) per year to describe long-time trends and seasonality; indicator variables for day of the week; natural cubic splines with 3 df at equally spaced quantiles for the average of dew point temperature at lag 0–1; linear terms for the average of ozone and CO at lag 0–1. These choices are motivated by several methodological and substantive papers on time-series analyses [21, 26, 27].

The effect of mean temperature has been investigated through the choice of two bases to describe the relationship in the space of temperature and lags; we illustrate a flexible model with natural cubic splines to describe the relationship in each dimension. The knots were placed at equally spaced values in the range of temperature, to allow enough flexibility in the tails, and at equal intervals in the logarithmic scale of lags, to allow more flexibility in the first part of the distributed lag curve, where more variability is expected [22, 28]. The maximum lag *L* was set to 30 days. Simpler models with the moving average of temperature in previous days have been fitted for comparison.

We have based the choice of the number of knots, which defines the df in each dimension, on modified Akaike and Bayesian information criteria for models with overdispersed responses fitted through quasi-likelihood [11, 27], given by:


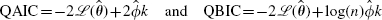
(13)

where ℒ is the log-likelihood of the fitted model with parameters 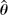 and 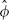 the estimated overdispersion parameter, whereas *k* and *n* are the number of parameters and number of observations, respectively. The best model is chosen that minimizes the criteria above.

All the analyses were performed with the software R, version 2.10.1 [8], using the package dlnm, version 1.1.1, developed by the first two authors and publicly available on the R comprehensive archive network (CRAN). The code of the main analysis is included in Appendix A.

### 5.2. Results

When used to compare different modelling choices, the QAIC led to a comparatively complex model, with 11 df for the space of the predictor and 5 df for the lag dimension, and a total of 55 parameters used to define the relationship. In contrast, the QBIC indicated a 5 × 5 df model, with 25 df spent to describe the overall effect. In the absence of any knowledge about the performances of these criteria within the DLNM framework, we chose the latter as our final model on the grounds of parsimony.

**Figure 1:**
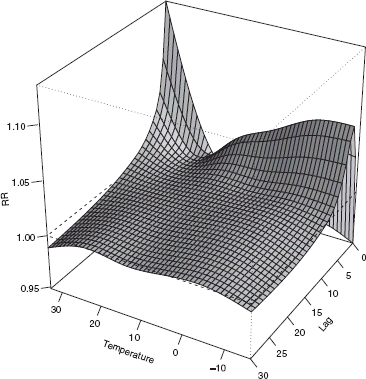
3-D plot of RR along temperature and lags, with reference at 21°C.

An overall picture of the effect of temperature on mortality is provided in Figure 1, showing a 3-D graph of the relative risk (RR) along temperature and lags compared with a reference value of 21°C, the point of overall minimum mortality. The plot shows a very strong and immediate effect of heat, and suggests a more delayed effect for extremely hot temperatures. The maximum effect of cold temperatures is reached approximately at lag 2–3. Inspection of the graph at longer lags suggests some harvesting for extreme temperatures.

**Figure 2:**
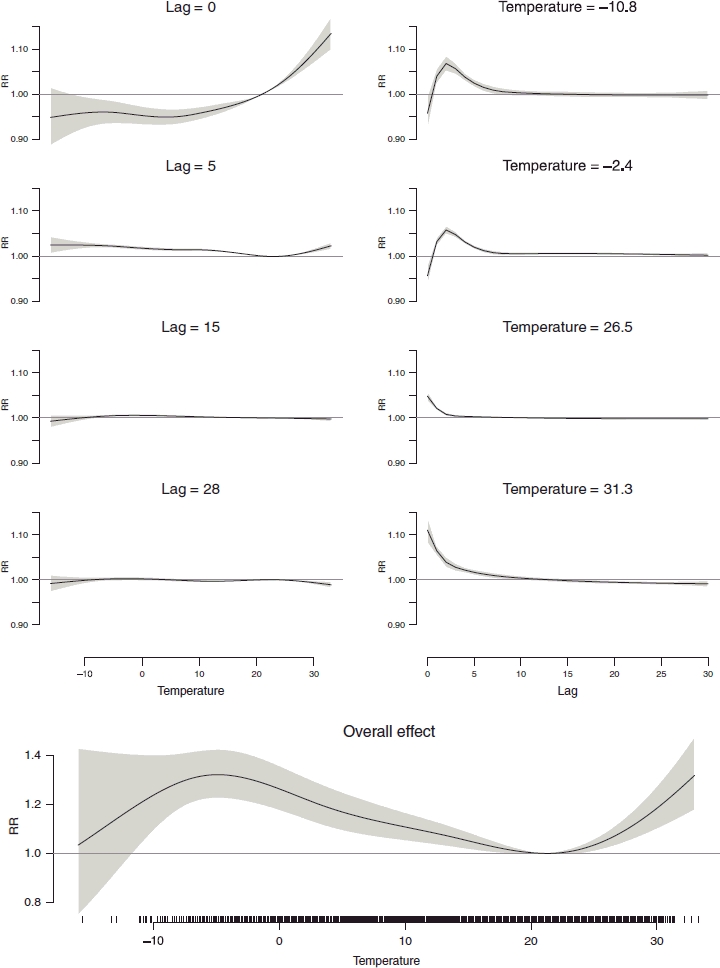
Plot of RR by temperature at specific lags (top left), RR by lag at 0.1th, 5th, 95th and 99.9th percentiles of temperature distribution (top right) and overall RR (below). Reference at 21°C.

Although the 3-D plot is a useful tool for summarizing the overall relationship in the two dimensions, uncertainty in the estimates cannot be included. In order to provide a more specific assessment of the relationship, we can plot the effects for specific temperatures or lags. Figure 2 shows the RR by temperature at specific lags (0, 5, 15 and 28) and by lag at specific temperatures (−10.8, −2.4, 26.5 and 31.3°C), corresponding approximately to 0.1th, 5th, 95th and 99.9th percentiles of temperature distribution (termed as moderate and extreme cold and heat). The overall effect of temperature, summing up the contributions for the 30 days of lag considered in the analysis, is included below. The shape of the temperature–mortality relationship seems to change along lags, with a different points of minimum mortality for lag 0 and 5 (first two graphs on top left). This plot confirms the more delayed effect of extreme heat if compared with moderate hot temperatures, with a significant risk lasting up to 10 and 3 days, respectively (third and fourth graphs from top right). Nonetheless, only extreme hot temperatures suggest a possible harvesting effect, starting after 15 days of lag. The overall estimated RR versus 21°C is 1.24 (95 per cent CI: 1.13–1.36) and 1.07 (95 per cent CI: 1.03–1.11) for extreme and moderate heat, respectively. Cold temperatures show a completely different pattern, with the effect of moderate cold sustained up to 25 days of lag (first two graphs on top right). In addition, the effect of cold seems to level off, with a slightly higher overall RR of 1.30 (95 per cent CI: 1.20-1.40) for moderate cold, compared to 1.20 (95 per cent CI: 1.04-1.39) for extreme cold (graph below).

To compare this DLNM with simpler alternatives, models with the moving average of lag 0–1 and lag 0–30 and the same spline functions for the space of temperature have been fitted. The former provides similar estimates for the effect of heat, but shows a weaker effect of low temperatures, with an estimated RR of 1.06 (95 per cent CI: 1.03–1.09) for moderate cold. This difference is probably due to underestimation, given the fact that low temperatures exert effects lasting longer than 2 days. Conversely, the moving average model with lag 0–30 shows similar effects for cold, but lower estimates for hot temperatures, with a RR of 1.01 (95 per cent CI: 0.97–1.04) and 1.06 (95 per cent CI: 0.97–1.17) for moderate and extreme heat, respectively. It is plausible that averaging over 31 days could cause some bias in the estimates, considering that each previous exposure within the lag period is assumed to provide the same contribution to the effect on each day. The criteria above indicate a better fit of DLNM, with a difference of 571 and 517 for QAIC and of 468 and 445 for QBIC if compared with lag 0–1 and 0–30 moving average models, respectively.

A sensitivity analysis has been carried out to assess the impact of model choices. In particular, we evaluate changes in the estimated overall effect (as described in the bottom of Figure 2) associated with varying the df used to specify the cross-basis functions (along both dimensions) and the seasonal and long-term trend component. Increasing the number of knots in the space of temperature produces a much less smoothed curve, probably due to overfitting, while no appreciable change is noted with different choices for spline in the lag dimension. Using more df to control for season and long-time trend does not affect the estimates, apart from a less pronounced decrease in the temperature–mortality curve at very low temperature. In addition, the inspection of lag and temperature-specific curves reveals that the supposed negative effect of heat at long lags, attributed to harvesting, completely disappears when increasing the seasonal control. This is plausible, given that the effects of models with an extended lag periods are more sensitive to the seasonal component.

## 6. Discussion

In this paper we have described the class of DLNMs, the members of which can be used to model the effect of factors showing at the same time non-linear dependencies and delayed effects. The specification of a DLNM is conceptually simple but flexible enough to allow a wide range of models including simple previously used as well as more complex new variants. The conceptual simplicity has allowed construction of an R package to fit this wide range of models.

One difficulty highlighted by this abundance of choice (basis types, number and placement of knots, maximum lag) is what criteria can be used to chose between alternatives. In the example above we used information criteria to guide choice of number of knots, but *a priori* arguments for choice of basis types and maximum lag. A previous discussion on choice of DLNM from an epidemiological perspective emphasized compromise between sufficient complexity to capture detail and sufficient simplicity to allow interpretation [7]. Because there is no consensus on what comprises an ‘optimal’ model, sensitivity analyses are particularly important, allowing dependence of key conclusions on model choice to be assessed. The broad range of DLMNs facilitates this. Regression diagnostics, such as residuals and partial autocorrelation plots, may also be helpful. In addition, we have discussed choice of DLNM assuming that it focuses on the variable of interest (temperature in our example). There is also a problem of model selection for covariates, some parts of which might also be DLNMs. This problem, sometimes referred to as adjustment uncertainty, has received some attention in time series studies of pollution [29, 30] as well as generally [31]. Again no consensus has emerged on what approach is optimal, and analyses of sensitivity to this component of model choice is also important.

The current implementation of DLNMs as illustrated in Section 5 is based on a completely parametric method, where the cross-basis dimension *v_x_* × *v_l_* equals the number of df spent to describe the relationship. Recently, interesting alternatives based on penalized regression with low-rank smoothers have been proposed to deal with non-linear effects [32, 33], and also applied to describe the distributed lag curve [6, 22]. Although completely parametric approaches seems to be preferred to control for season and long-term trend in time series data [27, 34, 35], the penalized methods could show some advantage in the bi-dimensional framework of DLNM. This issue represents an opportunity for further development, and could benefit from the research already carried out on penalized tensor-product smoothers [25, 36]. In addition, the algebraic definition in Section 4 is still valid in this new context, and only the estimation algorithm to derive 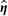 and 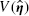 actually changes.

The development of DLNMs described in Section 4 involves only a comparatively complex parameterization of the lagged exposure series, as expressed by (7). Although our application has involved the use of an overdispersed Poisson log-linear model, we do emphasize through the development and notation in (1), that this framework has very general applicability, for example to time series data with other outcome distributions. More importantly, the main concept is fairly general, and can be easily translated in other study design and regression models.

The analysis of the data for New York during the period 1987–2000 offers some evidence for the potential of this framework to highlight complex dependencies of environmental factors, which would be largely obscured when using simpler models. We believe this approach represents a useful tool to gain understanding of phenomena investigated in environmental studies and other scientific fields.

## Appendix A: R code

The following code reproduces the main analysis and graphs included in Section 5. The packages dlnm and NMMAPSlite may be downloaded directly through R from the CRAN.

A detailed overview of the capabilities of the package dlnm is illustrated in the vignette included in the implementation, available typing vignette (“dlnmOverview”) in R.

require(dlnm);require(NMMAPSlite)
##############################
# LOAD AND PREPARE THE DATASET
##############################
initDB()
data <- readCity(“ny”, collapseAge = TRUE)
data <- data[,c(“city”, “date”, “dow”, “death”, “tmpd”, “dptp”, “rhum”, “o3tmean”, “o3mtrend”, “cotmean”, “comtrend”)]
# TEMPERATURE: CONVERSION TO CELSIUS
data$temp <- (data$tmpd-32)*5/9
# POLLUTION: O3 AND CO AT LAG-01
data$o3 <- data$o3tmean + data$o3mtrend
data$co <- data$cotmean + data$comtrend
data$o301 <- filter(data$o3,c(1,1)/2,side=1)
data$co01 <- filter(data$co,c(1,1)/2, side=1)
# DEW POINT TEMPERATURE AT LAG 0-1
data$dp01 <- filter(data$dptp,c(1,1)/2,side=1)
##############################
# CROSSBASIS SPECIFICATION
##############################
# FIXING THE KNOTS AT EQUALLY SPACED VALUES
range <- range(data$temp,na.rm=T)
ktemp <- range [1] + (range [2]-range [1])/5*1:4
# CROSSBASIS MATRIX
ns.basis <- crossbasis(data$temp,varknots=ktemp,cenvalue=21, lagdf=5,maxlag=3 0)
##############################
# MODEL FIT AND PREDICTION
##############################
ns <- glm(death ∼ ns .basis + ns (dp01, df=3 ) + dow + o301 + co01 +
ns(date,df=14*7),family=quasipoisson(), data) ns.pred <- crosspred(ns.basis,ns,at=-16:33)
##############################
# RESULTS AND PLOTS
##############################
# 3-D PLOT (FIGURE 1)
crossplot(ns.pred,label=“Temperature”)
# SLICES (FIGURE 2, TOP)
percentiles <- round(quantile(data$temp,c(0.001,0.05,0.95,0.999)), 1) ns.pred <- crosspred(ns.basis,ns,at=c(percentiles,-16:33)) crossplot(ns.pred,"slices”,var=percentiles,lag=c(0,5,15,28), label="Temperature”)
# OVERALL EFFECT (FIGURE 2, BELOW)
crossplot(ns.pred,"overall”,label="Temperature”, title="Overall effect of temperature on mortality
New York 1987–2000” )
# RR AT CHOSEN PERCENTILES VERSUS 21C (AND 95%CI)
ns.pred$allRRfit[as.character(percentiles)]
cbind(ns.pred$allRRlow,ns.pred$allRRhigh)[as.character(percentiles),]
##############################
# THE MOVING AVERAGE MODELS UP TO LAG x (DESCRIBED IN SECTION 5.2)
# CAN BE CREATED BY THE CROSSBASIS FUNCTION INCLUDING THE
# ARGUMENTS lagtype=“strata”, lagdf=1, maxlag=x
